# Operando analysis of a solid oxide fuel cell by environmental transmission electron microscopy

**DOI:** 10.1038/s41467-023-43683-4

**Published:** 2023-12-02

**Authors:** Q. Jeangros, M. Bugnet, T. Epicier, C. Frantz, S. Diethelm, D. Montinaro, E. Tyukalova, Y. Pivak, J. Van herle, A. Hessler-Wyser, M. Duchamp

**Affiliations:** 1 Photovoltaics and Thin-Film Electronics Laboratory (PV-Lab), École Polytechnique Fédérale de Lausanne (EPFL), Rue de la Maladière 71b, 2000 Neuchâtel, Switzerland; 2 Centre Suisse d’Electronique et de Microtechnique (CSEM), Jaquet-Droz 1, 2002 Neuchâtel, Switzerland; 3 Univ Lyon, CNRS, INSA-Lyon, UCBL, MATEIS, UMR 5510, 69621 Villeurbanne, France; 4 Univ Lyon, UCBL, IRCELYON, UMR CNRS 5256, F-69626 Villeurbanne, France; 5 Group of Energy Materials (GEM), École Polytechnique Fédérale de Lausanne (EPFL), Rue de l’Industrie 17, 1951 Sion, Switzerland; 6 SolydEra S.p.A., 38017 Mezzolombardo, Italy; 7 Laboratory for in situ & operando Electron Nanoscopy, School of Materials Science and Engineering, Nanyang Technological University (NTU), 50 Nanyang Avenue, 63737 Singapore, Singapore; 8 DENSsolutions, Informaticalaan 12, 2628 ZD Delft, The Netherlands; 9 MajuLab, International Joint Research Unit UMI 3654, CNRS, Université Côte d’Azur, Sorbonne Université, National University of Singapore, Nanyang Technological University, Singapore, Singapore

**Keywords:** Fuel cells, Transmission electron microscopy, Batteries, Materials for devices

## Abstract

Correlating the microstructure of an energy conversion device to its performance is often a complex exercise, notably in solid oxide fuel cell research. Solid oxide fuel cells combine multiple materials and interfaces that evolve in time due to high operating temperatures and reactive atmospheres. We demonstrate here that *operando* environmental transmission electron microscopy can identify structure-property links in such devices. By contacting a cathode-electrolyte-anode cell to a heating and biasing microelectromechanical system in a single-chamber configuration, a direct correlation is found between the environmental conditions (oxygen and hydrogen partial pressures, temperature), the cell open circuit voltage, and the microstructural evolution of the fuel cell, down to the atomic scale. The results shed important insights into the impact of the anode oxidation state and its morphology on the cell electrical properties.

## Abstract (web summary)

By contacting a solid oxide fuel cell to a microelectromechanical system inside an environmental electron microscope, the authors establish links between environmental conditions (gas atmosphere, temperature), cell voltage and atomic-scale structure.

## Introduction

Improving the performance of energy conversion technologies often requires inputs provided by characterisation techniques that can bring detailed insights concerning the crystallography, chemistry, and microstructure of materials. However, these microstructural analyses are usually performed ex situ, i.e., in conditions that differ from the ones experienced by the materials in a functioning device, where the latter is exposed to a gas atmosphere, elevated temperature, electrical bias, etc. These conditions often lead to microstructural alterations that are missed when characterising the sample ex situ, hence complicating the understanding of structure-property links. The analysis of the microstructure of materials during device operation is particularly challenging for solid oxide fuel cells (SOFCs) and their solid oxide electrolysis cells (SOECs) counterparts. Their harsh operating conditions combining high temperatures (600–1000 °C to ensure sufficient ionic conductivity of the electrolyte)^1^, reducing and oxidising gases (typically H_2_ and O_2_ or air), and electrical bias are difficult to recreate within characterisation setups.

Overall, SOFCs convert the chemical energy of a fuel directly into electricity through an electrochemical process or, vice versa, electricity into usable and storable fuels via SOECs^2,3^ with a high performance and with negligible emissions of NO_x_ or SO_x_. Standard SOFC/SOEC designs include an yttria-stabilised zirconia (YSZ) electrolyte sandwiched between a thick nickel (Ni)/YSZ anode support and a cathode based on strontium-doped lanthanum manganite (LSM) or lanthanum strontium cobalt ferrite (LSCF), the latter requiring the use of a ceria diffusion barrier^2,4^. For the anode, NiO is typically co-sintered with YSZ and then reduced to its metallic Ni active state during the first operation of the cell. The volume loss associated with this reduction reaction leaves pores in the anode, ensuring a permeation of the fuel to the electrochemically active sites, the triple-phase boundaries (TPBs, Ni-YSZ-porosity in the anode)^5^. The high operating temperatures may then trigger various degradation mechanisms, which will eventually lower the operational performance of the system^6^. Indeed, TPBs in both anode and cathode (LSM-YSZ-porosity) may become deactivated as a result of various mechanisms, e.g., due to poisoning by chromium or sulfur^7–11^, or due to a reorganisation of the Ni catalyst through coarsening or reduction-oxidation (redox) cycling^5,12–14^.

The understanding of these degradation mechanisms has benefited from microstructural insights retrieved in situ or even *operando*, i.e., by raising the sample temperature in a relevant gas atmosphere directly in the characterisation apparatus, and, for *operando* studies, in conjunction with electrically monitoring the sample. More specifically, X-ray photoelectron spectroscopy has been used to monitor surface chemistries and surface potentials of SOFCs, also under electrical polarisation^15–17^. The anode crystallographic properties^18,19^ and internal stress^20,21^ have been characterised using X-ray diffraction during reduction-oxidation cycles. X-ray absorption near edge structure studies have focused on the identification of oxidation states^22,23^, while X-ray tomography has enabled a three-dimensional assessment of microstructural changes depending on environmental conditions^24,25^. Optical techniques such as Raman spectroscopy have also been used, either in situ or *operando*, to investigate the composition, microstructure, surface temperature, or presence of adsorbed surface species in various temperature and gas conditions^26–31^. Thermal imaging has been combined with electrical measurements to study sulphur contamination^32^. In another example, the mobility of Ni on YSZ has been monitored *operando* using confocal laser scanning microscopy^33^.

While providing valuable insights, the methods listed above are limited to a spatial resolution typically in the tens of nanometre range at best, meaning that structural details occurring at finer length scales remain elusive. Electron microscopy is one of the few techniques with the ability to retrieve microstructural, chemical and crystallographic properties down to the (sub-)nanometre, also in situ by raising the sample temperature in a gas atmosphere directly inside the microscope^34–41^. Indeed, a spatial resolution below 0.1 nm has been reached with modern aberration-corrected environmental transmission electron microscopy, which has enabled the direct observation of metal sintering^42^, metal nanoparticles-support interactions^43^, surface reconstructions^44^, atomic-scale dynamics^45,46^, or phase transformations^47^. Regarding SOFC/SOEC research, environmental transmission electron microscopy coupled to various spectroscopies has enabled detailed investigations of the reduction and reoxidation pathways of the Ni catalyst^39–41,48–50^. However, one limitation of such environmental transmission electron microscopy experiments is that the electrical properties of the SOFC/SOEC sample are not recorded due to the complexity of electrically contacting the thin “electron-transparent” sample. This in turn complicates the understanding of structure-property links.

Capitalizing on recent advances in focused ion beam (FIB) sample preparation protocols^51–54^, in microelectromechanical systems (MEMS) for combined heating and biasing studies inside microscopes^55,56^, and in environmental transmission electron microscopy techniques^57^, we demonstrate here that SOFCs/SOECs can be analysed *operando* by environmental transmission electron microscopy by recording simultaneously electrical and microstructural properties in (adapted) operational conditions. A single-chamber configuration, i.e., where the entire SOFC sample and both the fuel and the oxidant gas are present in the same chamber, is selected to avoid the need to constrain the oxidant gas to the cathode and the fuel to the anode^38,58^. The functioning mechanism of single-chamber SOFCs remains similar to that of conventional dual chamber systems, with the exception that the anode and cathode need to exhibit selectivity towards either the fuel oxidation or the oxidant gas reduction reactions, respectively. Here, the open circuit voltage building up across thin lamellae composed of the anode, electrolyte and cathode is measured as a function of the O_2_-to-H_2_ ratio at elevated temperature, while monitoring microstructural properties down to the atomic scale. These experiments open new perspectives for the analysis of the links between performance and microstructure of SOFCs/SOECs and in energy materials in general.

## Results and discussion

### Single-chamber experimental setup and SOFC anode activation

**Fig. 1:**
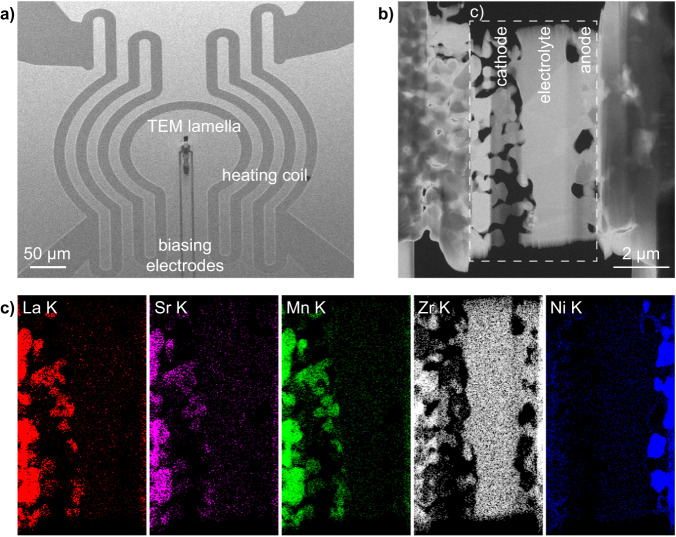
TEM lamella of a SOFC mounted on a MEMS with heating and electrical biasing/monitoring capabilities. **a** Scanning electron microscopy (SEM) image of a MEMS chip for operando transmission electron microscopy (TEM). The anode and cathode of the SOFC lamella are electrically connected to the biasing electrodes of the MEMS. **b** STEM ADF micrograph of the electrically connected SOFC sample, and **c** corresponding STEM EDX maps of the K edges of the main elements present in the initial SOFC device acquired from the dashed area shown in **b**.

The cell architecture investigated here consists of an LSM/YSZ cathode, a YSZ electrolyte and a NiO/YSZ anode, as shown in Fig. 1. To ensure industrial relevance, the SOFC investigated here was produced by SolydEra S.p.A. using tape-casting. The electrolyte was made thinner (2 µm) than usual to enable the fabrication by FIB of a TEM lamella containing all the relevant interfaces of the cathode-electrolyte-anode cell. The lamella was contacted to a MEMS chip from DENSsolutions with simultaneous heating and biasing capabilities (Fig. 1a, see Materials and Methods for details). The TEM lamella was mounted onto a prototype DENSsolutions MEMS holder and inserted in the column of a FEI Titan G2 environmental transmission electron microscope (ETEM). A scanning TEM (STEM) annular dark-field image (ADF) image of the as-prepared SOFC is shown in Fig. 1b. Corresponding elemental maps obtained by STEM energy dispersive X-ray spectroscopy (EDX) are displayed in Fig. 1c, highlighting how the different phases are distributed in the initial sample. The YSZ electrolyte is dense with grains of about 1–2 µm, while the LSM/YSZ cathode is porous to ensure oxygen access to the TPBs (LSM-YSZ-porosity on the cathode side). On the other hand, the NiO/YSZ anode precursor is dense in its as-sintered state.

Prior to operation, the as-sintered NiO phase needs to be reduced to Ni, its active state. The porosity that will result from the process will also enable to form TPBs on the anode side^5^. To activate the Ni catalyst of the cermet, 10 to 15 mbar of forming gas (5 v/v% of H_2_ in N_2_) was introduced in the ETEM, a pressure approaching the maximal pressure allowed in the environmental chamber. The temperature was increased up to 750 °C to trigger the reduction of NiO to Ni. STEM ADF micrographs acquired at various temperatures and pressures are given in Fig. S1, highlighting how the microstructural changes occur in the anode during the activation of the Ni. The reduction reaction becomes visible through the creation of pores within the NiO grains, with pores forming preferentially at the interfaces with YSZ due to a quick coarsening of the Ni phase at these temperatures. As highlighted in prior studies^39, 40,48,50^, the NiO reduction kinetics is slow inside the ETEM in these flow and pressure conditions. The reaction rate is initially controlled by the nucleation of the first Ni seeds. The presence around the reaction sites of H_2_O released by the reduction then likely slows down the reaction rate at high conversion fractions. On the other hand, the cathode remains unchanged in forming gas up to this temperature of 750 °C and within this time scale of 210 minutes (Fig. S2).

### H_2_-to-O_2_ gas ratio, SOFC voltage and microstructure

To trigger the operation of the SOFC lamella in a single-chamber configuration, the temperature was lowered to 600 °C. In addition to limiting thermal stress, this temperature was selected in this single-chamber configuration to limit the activity of the LSM cathode towards the fuel oxidation reaction^59^, while still ensuring the activity of the Ni/YSZ anode towards this reaction (>550 °C)^60^. The forming gas H_2_/N_2_ flow was set to 3 ml/min before introducing an additional flow of O_2_ of ~0.1 ml/min, leading to an increasing O_2_-to-H_2_ ratio in the ETEM. Note that the O_2_ flow was set to the minimum value allowed by the Brooks mass flow controller ahead of the ETEM. In these conditions, the total pressure in the ETEM chamber reached 15.8 mbar.

**Fig. 2:**
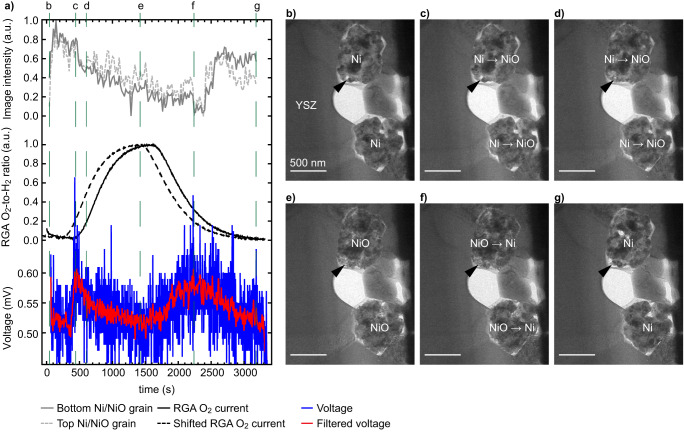
O_2_-to-H_2_ ratio and its impact on the SOFC voltage and microstructure. **a** Plots showing the evolution with time of the average TEM image intensity measured at the location of two Ni grains, the ratio of the residual gas analyser (RGA) O_2_ and H_2_ signals (raw data, full line, and curve shifted forward by 180 seconds, dashed line), and the voltage measured between the two biasing electrodes (raw data in blue and after the application of a gaussian filter in red, see supplementary information for details). **b**–**g** Selection of corresponding TEM images of the two Ni grains located next to the yttria-stabilised zirconia (YSZ) electrolyte, the intensity of which is reported in **a**, taken at critical steps of the oxidation and reduction processes. Arrowheads highlight morphological changes occurring during the oxidation and then reduction of Ni, see text for details.

We investigated the impact of a varying O_2_-to-H_2_ ratio and monitored the cell open circuit voltage (OCV) in relation to the morphology of the Ni catalyst (Fig. 2). Note that several reduction-oxidation cycles took place between Figs. S1 and 2. Figure 2a plots i) the evolution with time of the average TEM image intensity of the two Ni grains shown in Fig. 2b–g (i.e., intensity of the image averaged over the area of the two grains), ii) the ratio between the O_2_ and H_2_ signals obtained from the residual gas analyser (RGA) appended to the exit of the ETEM chamber, and iii) the OCV between the two MEMS biasing electrodes (see Fig. 1a). The as-measured RGA O_2_-to-H_2_ ratio data (full line) was advanced by 180 seconds to correct for the time needed by the gas to travel from the reaction chamber to the RGA (dashed line, see Materials and Methods section for details). Figure 2b–g shows a selection of TEM images detailing the evolution of the two Ni grains, the intensities of which are plotted in Fig. 2a. The full sequence of TEM images of Fig. 2 is available online (10.5281/zenodo.8414459). From Fig. 2a, a direct correlation between Ni grain average intensity, presence of O_2_, and OCV between the anode and cathode is noticed. When introducing O_2_ in the ETEM chamber, the image intensity remains constant for about 500 s, which coincides with a small increase in OCV between the MEMS electrodes. As the O_2_-to-H_2_ ratio increases further (from 600 s to 1500 s), the OCV drops rapidly back to a value close to its initial baseline, while the Ni grains become darker. This lowering of the TEM image intensity is indicative of an oxidation of the Ni grains to NiO: oxygen is incorporated in the Ni grains, leading to a thickening of the grains and to the filling of voids (see arrowheads in Fig. 2b–d), which in turn decreases the number of electrons collected by the TEM camera due to additional scattering to high angles. This oxidation of the Ni catalyst in the TEM images is confirmed by tracking the evolution of electron energy-loss spectra (EELS) of the Ni-L_2,3_ edges (Fig. S3). The increasing intensity of the Ni-L_3_ edge (~855 eV) with respect to the L_2_ edge (~872 eV) indicates an oxidation of Ni during the first part of the experiment^61^. As discussed elsewhere^39,49,62^, this volume expansion of Ni upon oxidation is larger than that predicted by the Pilling-Bedworth ratio due to unbalanced mass transport mechanisms. In this temperature range <1000 °C, Ni^2+^ ions diffuse outwards through the NiO scale grain boundaries faster than O^2-^ ions diffuse inwards, leading to the injection of vacancies at the Ni/NiO interface and eventually to the formation of internal voids within the growing NiO scale. When stopping the O_2_ flow at ~1500 s, a delay of several minutes (until ~2300 s) is observed before the image intensity starts to increase again as the NiO grains reduce back to Ni (Fig. 2f). The Ni grains shrink during the reduction reaction and porosity re-appears within these grains (see arrowheads in Fig. 2e–g). In parallel, the OCV starts to increase when O_2_ is removed after ~1500 s, before decreasing from ~2500 s onwards. The OCV increases and decreases at a slower rate compared to the first peak (when O_2_ was introduced in the chamber).

### Correlation between Ni oxidation state and SOFC voltage

**Fig. 3:**
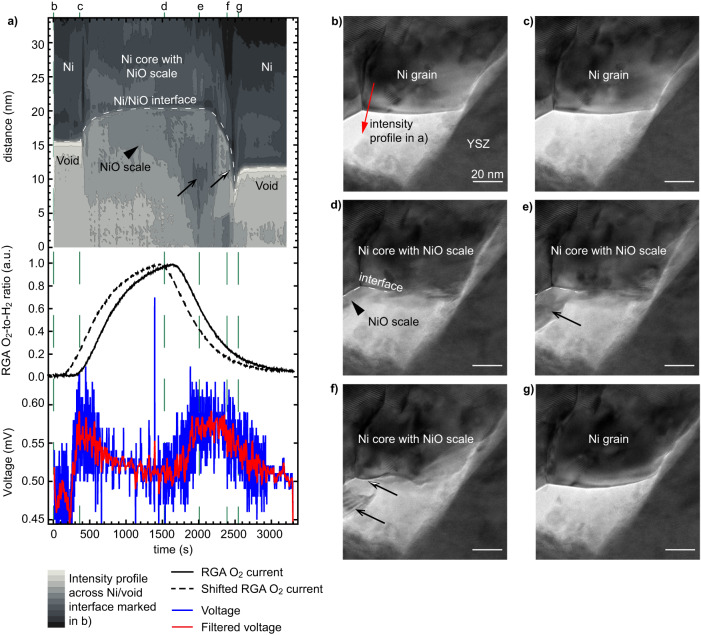
Oxidation and reduction cycling of a Ni grain depending on the O_2_-to-H_2_ ratio and resulting voltage measured across the SOFC sample. **a** Contour plot of the evolution of the TEM image intensity taken along the arrow shown in **b**, the residual gas analyser (RGA) O_2_-to-H_2_ ratio (raw data, full line, and shifted forward by 180 seconds, dashed line), and open circuit voltage measured between the anode and cathode (raw and gaussian-filtered data). **b**–**g** Selection of TEM images of the edge of a Ni grain at the critical steps of the reoxidation and reduction processes. Black arrows and arrowheads highlight key morphological changes occurring at the surface of the Ni grain, as discussed in the text.

**Fig. 4:**
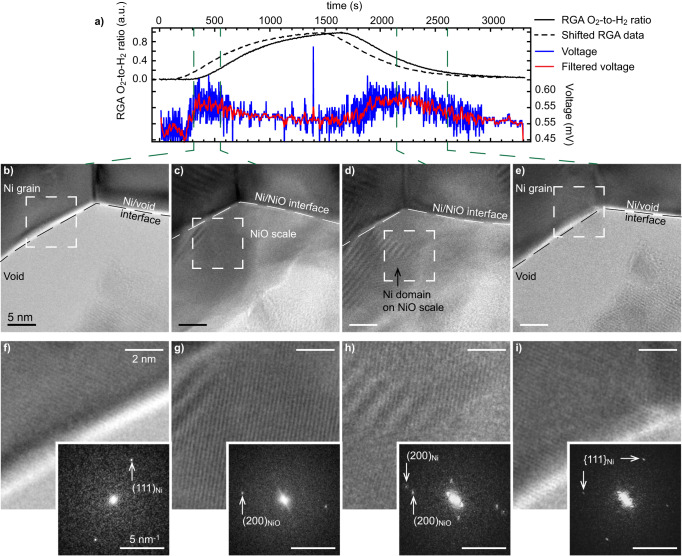
Atomic-scale imaging of a Ni grain as a function of the O_2_-to-H_2_ ratio. **a** Residual gas analyser (RGA) O_2_-to-H_2_ ratio (raw data, full line, and shifted forward in time by 180 seconds, dashed line), and open circuit voltage measured between the anode and cathode (raw and gaussian-filtered data). **b**–**e** High-resolution TEM images of the edge of a Ni grain at the critical steps of the reoxidation and reduction processes, and **f**–**i** Fourier-filtered magnified micrographs and corresponding FFTs taken from the dashed regions in **b**–**e**. The interface of the Ni grain, with a void or with the NiO scale, is marked by a dashed line in **b**–**e**.

From Fig. 2c and the EELS data of Fig. S3, it appears that the first increase in OCV is correlated with the presence of Ni in its metallic state (smaller volume, compact morphology with some open porosity as shown by the arrowhead, lower Ni L_3_/L_2_ EELS ratio). To rationalise the OCV variations observed in Fig. 2 and explain the second OCV peak, similar sequences capturing the oxidation and reduction of the Ni catalyst were performed at higher spatial resolution. Figure 3 details the morphological changes occurring at the surface of one Ni grain during an oxidation and then a reduction. Figure 3a shows the evolution in time of an intensity profile taken across a Ni/void interface, which is shown in the form of a contour plot (taken from the region marked by an arrow in Fig. 3b). The RGA and OCV data are also plotted in Fig. 3a. A first increase in OCV is observed after ~380 s, which coincides with the presence of both O_2_ and H_2_ in the ETEM chamber and with Ni in its metallic state (as in Fig. 2). Indeed, the intensity profile taken at the surface of one Ni grain does not change during these early stages, despite the (low) O_2_ partial pressure now being present in the chamber (Fig. 3a). The dense Ni grain morphology remains identical between Fig. 3b, c. As the O_2_-to-H_2_ ratio increases after 400 s of experiment, a NiO scale starts to form on the metallic Ni grain (Fig. 3d). The surface of the Ni grain retracts towards the centre of the Ni grain (see dashed line marking the Ni/NiO interface in Fig. 3a, d, e). The Ni grain is now covered by a NiO scale that expands outwards. The TEM image intensity within the region that was previously a void now decreases as NiO is now forming there (arrowhead in Fig. 3a, d). Once the O_2_ flow is stopped and the O_2_-to-H_2_ ratio starts to decrease after 1500 s, the position of the Ni/NiO interface stops retracting and remains immobile along the y axis of the line profile. In parallel, the intensity at the location of the NiO scale starts to decrease further (see black arrow in Fig. 3a, e). As it will be confirmed below in Fig. 4, this loss in intensity results from the growth of new Ni domains directly on the NiO scale as the O_2_-to-H_2_ ratio decreases. The OCV increases when Ni is present on the outer surface of the NiO scale. Furthermore, Ni L_3_,_2_ EELS data shown in Fig. S3 is consistent with such a mixed NiO/Ni system: an intermediate L_3_/L_2_ ratio is measured in these conditions. After 2200 s, the Ni/NiO interface is observed to move downwards as the NiO scale disappears and the Ni islands present on the scale surface merge with the centre of the Ni grain which did not fully reduce (see half black half white arrows in Fig. 3a, f). In Fig. 3g, the NiO scale has completely disappeared. The second voltage increase from ~1500 s to ~2300 s is broader than the first one. The full dataset used to make Fig. 3 is available online (10.5281/zenodo.8414459).

To verify that the second OCV increase coincides with the nucleation of Ni islands on the NiO scale, higher magnification images of the interface analysed in Fig. 3a are reported in Fig. 4. Lattice fringes can be periodically resolved, enabling an indexation of the different phases depending on the environmental conditions. At low O_2_-to-H_2_ ratios, fast Fourier transforms (FFT) reveal that the presence of Ni (111) reflections coincides with the first OCV increase observed after ~400 s (Fig. 4a), in agreement with previous interpretations. When reaching higher O_2_ partial pressures, new crystalline domains form on the surface of the Ni grains 550 s after the start of the experiment. Lattice fringes with the same lattice spacing (~4.7 nm^−1^) but with a different orientation than the parent Ni grain can be resolved: these are attributed to NiO (200) planes (Fig. 4c, g). In addition, faint reflections that correspond to NiO (111) planes can also be detected (4.1 nm^−1^). At this point, the OCV starts to drop, which is consistent with an oxidation of Ni. The O_2_ flow was then stopped before reaching a full oxidation of the Ni grains. After an incubation time (from 1500 s to 2150 s), Ni (200) reflections (5.3 nm^−1^) start to appear on the NiO scale (Fig. 4d, h). This observation confirms the presence of Ni islands on the NiO scale in these intermediate O_2_-to-H_2_ ratio conditions, which coincide with an increase in OCV. The NiO scale then becomes fully reduced after 2600 s as the O_2_-to-H_2_ ratio decreases: the Ni islands present on the surface eventually merge with the parent Ni grain and the OCV drops back to its baseline value (Fig. 4e, i). As it was not fully oxidised, the Ni grain keeps its initial orientation after one partial oxidation and reduction cycle. All the images of the experiment linked to Fig. 4 can be found online (10.5281/zenodo.8414459).

From Figs. 2–4, the increase in OCV of the single-chamber SOFC coincides with the presence of both metallic Ni on the outer surface of the anode and a small partial pressure of O_2_ in the chamber. When one of these two criteria is not fulfilled the OCV measured across the cell drops back to its baseline value. It should be noted that the minimum flow allowed by the mass flow controllers coupled with the maximum pressure achievable in the ETEM chamber limited us to transient experiments: a constant O_2_-to-H_2_ ratio maintaining the Ni reduced in the anode side and sufficient O_2_ to have a constant OCV could not be reached in the ETEM. In other words, the thin lamella oxidised quickly upon O_2_ exposure. These oscillations triggered by the oxidation of the Ni anode have also been reported in literature when studying bulk systems^58^. Another observation is that the baseline voltage value of about 0.55 mV measured here does not depend on the gas atmosphere: it is similar in H_2_/N_2_ and in O_2_-rich atmospheres.

### Single-chamber operation of standard SOFCs

The same cathode-electrolyte-anode cell was studied ex situ in a single-chamber configuration at 600 °C. The difference is that these ex-situ measurements performed in an oven involved atmospheric pressures and 14-mm diameter button cells instead of pressures of a few mbar and µm^3^ lamellae as in the ETEM. The results shown in Fig. S4a reveal trends that are qualitatively similar to those observed in situ in the ETEM. Starting from a Ni/YSZ anode in a H_2_ atmosphere, a first peak in OCV occurs at intermediate O_2_-to-H_2_ ratios (0.5) shortly after adding O_2_ in the chamber. The OCV then decreases at higher O_2_ partial pressures, likely as a result of the oxidation of the Ni catalyst. After decreasing the O_2_ flow midway through the experiment, another OCV peak appears when back to intermediate O_2_-to-H_2_ ratios. Finally, the OCV decreases sharply as O_2_ is fully removed from the chamber. One key difference with environmental transmission electron microscopy experiments is that the OCV measured ex situ reaches 0.8 V, a standard value for (single-chamber) SOFCs^58^. Furthermore, from the ex-situ tests shown in Fig. S4 and as-expected, the OCV is maximum around the stoichiometric O_2_-to-H_2_ ratio of 0.5.

The OCV forming between the cathode and anode of a SOFC depends on the difference between the oxygen partial pressures at the cathode and anode, ${P}_{{O}_{2,{cathode}}}$ and ${P}_{{O}_{2,{anode}}}$, respectively. The OCV is defined by the Nernst equation ${OCV}=\frac{{RT}}{{nF}}{{{{\mathrm{ln}}}}}(\frac{{P}_{{O}_{2,{cathode}}}}{{P}_{{O}_{2,{anode}}}})$, where *R* is the ideal gas constant, *T* the temperature, *n* the number of electrons involved in the reaction, and *F* the Faraday constant^58^. In a single-chamber SOFC, this difference in ${P}_{{O}_{2}}$ results from the difference in selectivity between the electrodes: the anode needs to favour the partial oxidation of the fuel (${{{\rm{H}}}}_{2}+{{{\rm{O}}}}^{(2-)} \leftrightarrow {{{\rm{H}}}}_{2} {{{\rm{O}}}}+{{{\rm{2e}}}}^{-}$, reversible reaction at OCV), while the cathode should promote the electrochemical reduction of the oxygen (${\frac{1}{2}O}_{2}+{2e}^{-} \leftrightarrow O^{2-}$ at OCV). Based on the Nernst equation, the small OCV gains over the baseline measured in the ETEM of 0.1 mV correspond to a difference in ${P}_{{O}_{2}}$ between cathode and anode of 0.5%. While this value is orders of magnitude below the OCV measured ex situ (0.8 V), only few TPBs are present in the thin TEM lamella to create this difference in ${P}_{{O}_{2}.}$ Furthermore, pressure conditions differ by several orders of magnitude when comparing experiments performed in the ETEM to the ones at atmospheric pressure in the oven.

To evaluate whether this difference in ${P}_{{O}_{2}}$ between anode and cathode could also result from the full oxidation of the fuel directly in the anode (${H}_{2}+{\frac{1}{2}O}_{2}\leftrightarrow {H}_{2}O$) with the cathode remaining inactive, we measured in an oven the OCV of an electrolyte-anode half-cell contacted using a Au mesh as a function of the O_2_-to-H_2_ ratio (Fig. S4b). The half-cell system exhibits a different behaviour than the full cell: a single increase in OCV is observed initially at increasing O_2_-to-H_2_ ratios, followed by a slow OCV decay as the O_2_-to-H_2_ ratio is kept constant and then decreased back to 0. When ramping up the O_2_ flow to reach the stoichiometric O_2_-to-H_2_ ratio of 0.5, it appears that the first peak in OCV occurs irrespective of the presence of the cathode. On the other hand, the second increase in cell OCV previously observed with full cells is not measured with the half-cell system. The cell OCV becomes negative towards the end of the experiment, indicating that the anode eventually starts to act as a cathode with respect to the Au electrode in these conditions^63^. Such negative voltages are typically observed when NiO reduces to Ni (Fig. S5). As Au is inert towards the fuel^64^, the absence of such negative OCV values in experiments performed with full cells indicate that the cathode is active and contributes to the overall electrochemical reaction, at least for the second OCV peak. It should be noted that the constrictivity and tortuosity of the different components of the full cells or half button cells affect the OCV trends measured ex situ (see Fig. S6 for cross-section SEM image of the button cell). Indeed, the oxidation state of the Ni phase will evolve in time throughout the anode as a function of the gas atmosphere, which will affect the permeation of the different gases within the anode (as the Ni is oxidising/reducing) and in turn the OCV (see Fig. S7 and associated explanation). The effect is absent in environmental transmission electron microscopy experiments due to the difference in the geometry of the samples under test (thin lamellae with large free surfaces versus bulk samples).

### Single-chamber SOFC operation in the ETEM

Finally, to evaluate further the activity of the cathode directly in the ETEM, the current-voltage characteristics of a full cathode-electrolyte-anode cell were measured as function of the gas atmosphere composition (Fig. S8). For these experiments, a thin TEM lamella featuring the full cathode-electrolyte-anode cell was connected to a sourcemeter instead of a voltmeter. The results should be analysed with care as currents in the pA range are measured. The electron beam was hence blanked during these experiments to avoid impacting the *J-V* data. Variations in O_2_-to-H_2_ ratio are found to affect the overall current-voltage trends. Starting with Ni in its metallic state in a reducing atmosphere as in Figs. 2–4, the introduction of a small flow of O_2_ in the ETEM chamber leads current-voltage characteristics qualitatively similar to those measured with bulk SOFCs, with the thin lamella delivering what appears to be a small power in these conditions. At longer O_2_ exposures, Ni fully oxidises to NiO and the system becomes highly resistive and hence unable to “generate” any power. Combined with the ex-situ analyses detailed above comparing full and half cells, these current-voltage measurements obtained here in the ETEM indicate that the thin lamella seems to be functioning inside the microscope, meaning that both the cathode and anode are active.

**Fig. 5:**
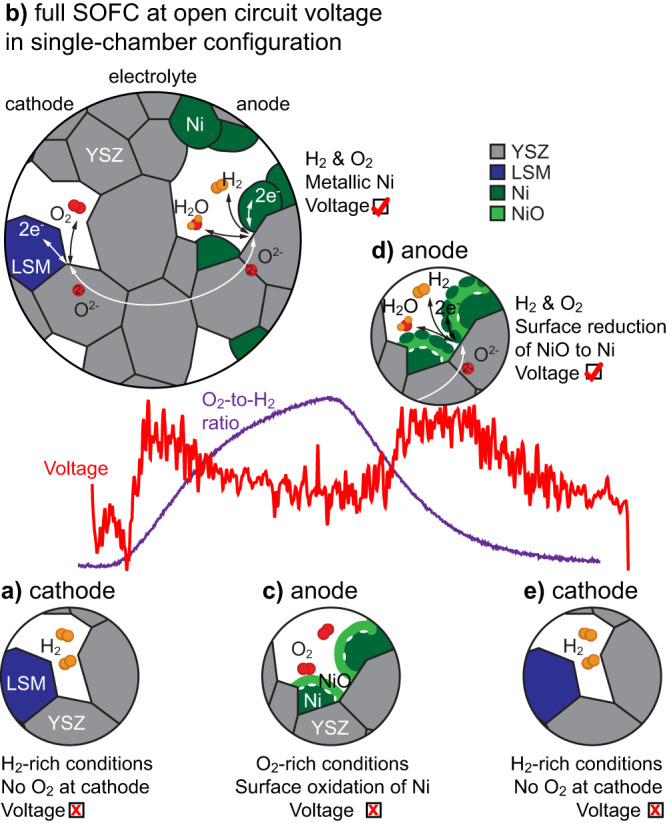
Schematic summary of the operation of the SOFC in a single-chamber configuration as observed operando in the ETEM. **a** At low O_2_-to-H_2_ ratios, the absence of O_2_ prevents its reduction at the cathode. **b** When introducing O_2_, the cell starts to deliver a voltage synonym of its operation until (**c**) the surface of the Ni grains oxidises. **d** When decreasing the O_2_-to-H_2_ ratio, the surface of the NiO scale starts to reduce into Ni islands, re-initiating the oxidation of the fuel at the anode, which results in a voltage increase. **e** The process stops at low O_2_-to-H_2_ ratios (as in **a**). YSZ stands for yttria-stabilised zirconia, LSM for lanthanum strontium manganite.

Figure 5 rationalises the *operando* observations obtained inside the ETEM. Starting with the Ni catalyst in its metallic electrochemically active state, the OCV remains low at small O_2_-to-H_2_ ratios as the O_2_ electrochemical reduction reaction at the cathode side is inhibited due to the absence of O_2_ (Fig. 5a). At increasing O_2_-to-H_2_ ratios, a small yet measurable OCV gain is measured (Fig. 5b). While the full oxidation of the fuel directly on the anode may contribute to this first OCV gain, the difference in selectivity between the anode and cathode also ensures that some of the O_2_ reduces at the cathode and some of the H_2_ oxidises partially at the anode. As the O_2_-to-H_2_ ratio continues to increase, the Ni catalyst starts to oxidise on its surface, which inhibits the adsorption and dissociation of H_2_, and hence stops the electrochemical reaction (Fig. 5c). When the O_2_-to-H_2_ ratio decreases, Ni islands start to nucleate on the NiO scale, leading to the formation of electrochemically active sites in the anode and to a second OCV gain (Fig. 5d). The NiO scale then completely reduces to metallic Ni as the O_2_-to-H_2_ ratio decreases further. Below a certain O_2_-to-H_2_ ratio threshold, the partial pressure of O_2_ is insufficient to sustain the oxygen reduction reaction in the cathode and the H_2_O formation reaction stops (Fig. 5e).

Overall, we demonstrated here that a SOFC can be analysed *operando* in a single-chamber configuration by environmental transmission electron microscopy. Both H_2_ and O_2_ were introduced in the microscope chamber, whilst keeping the cell at high operating temperature (600 °C) and observing its microstructure down to the atomic scale. By varying the O_2_-to-H_2_ ratio, direct correlations between cell OCV, gas atmosphere and microstructure of the Ni catalyst were established. At intermediate O_2_-to-H_2_ ratios and when the Ni catalyst is maintained in its metallic state, a small yet distinct gain in OCV between the two electrodes of the thin FIB-prepared lamella is measured. Based on the comparison with ex-situ experiments and current-voltage measurements obtained in situ in the microscope, the OCV that builds up in these conditions appears to result at least partially from the difference in selectivity between the anode and cathode for the partial fuel oxidation and oxidant gas reduction reactions, respectively. Depending on O_2_-to-H_2_ ratio, the surface oxidation of Ni stops the fuel oxidation reaction, while the growth of Ni islands on the NiO scale restarts it. Looking ahead, such *operando* experiments in the ETEM should enable to investigate a wide range of degradation pathways affecting SOFCs/SOECs, notably the poisoning of electrochemically active TPBs of both cathode and anode, or the impact of a coarsening of the Ni catalyst.

## Methods

FIB samples for *operando* characterization were prepared using a ZEISS Crossbeam 540 and contacted to a double-tilt 6 contacts DENSsolutions TEM holder and eventually to a voltmeter. The FIB-prepared samples were thinned once on the MEMS chip using a final voltage of 5 kV to reduce Ga^+^-induced damage and possible Ga^+^-rich surface short-circuits, which are particularly detrimental to biasing experiments. The thickness of the TEM lamella was ~200 nm. TEM experiments were performed in an image-Cs-corrected environmental FEI Titan microscope operated at 300 kV equipped with a CMOS camera (Gatan Oneview), a solid state EDX detector (Oxford 80), and an electron energy-loss spectrometer (Gatan Tridiem 965 ER). Analysis involved STEM imaging using an ADF detector, high-resolution TEM imaging, EDX and EELS. TEM movies were recorded using a home-made Gatan Digital Micrograph script, which blanks the electron beam between image acquisitions to avoid any contribution of secondary electrons to the measured voltage and beam-induced artefacts. TEM micrographs were acquired here every 20 seconds. A mass spectrometer (Pfeiffer Vacuum Model PrismaPlus™ QMG 220) located at the exit of the ETEM chamber was used to quantify the O_2_-to-H_2_ gas ratio by following the mass-to-charge ratios of 32 and 2 for O_2_ and H_2_, respectively. The time taken by the gas to reach the RGA was estimated by monitoring the delay between the introduction of the gas and its detection by the RGA. Data from the voltmeter was filtered: outliers induced by the periodic presence of the electron beam (every 20 seconds) were removed using a homemade *Mathematica* script, before filtering the resulting data using a gaussian filter spanning across 4 data points. EELS acquisitions were carried out in STEM mode using the spectrum imaging approach implemented in *Gatan Digital Micrograph*. The spectra were background-subtracted using a power law function, aligned with respect to the Ni-L_3_ energy at ~855 eV, and spectra were normalised on the Ni-L_2_ edge to highlight the evolution of the L_3_/L_2_ intensity ratio. The TEM lamellae were relatively thick (thickness/electron mean free path ≥ 1) to maintain the structural integrity of the SOFC, however preventing a precise quantification of the Ni oxidation state. Current-voltage measurements were performed in the environmental FEI Titan TEM by contacting the MEMS chip to a sourcemeter. The voltage was swept from −0.04 V to +0.04 V to avoid passing a high current density through the thin lamella. Scanning electron microscopy images were acquired with a Zeiss Gemini 2 with an acceleration voltage of 3 kV and a beam current of 300 pA.

Ex-situ tests were performed using a 14-mm diameter button cell featuring the same materials from the same batch as those tested by *operando* environmental transmission electron microscopy or electrolyte-anode half cells made of similar Ni/YSZ materials but from a different batch. The button cells were pressed between two gold meshes and placed inside a vertical furnace (Rohde, TE 10 Q SEV). The gas composition was adjusted by mixing individual gases. Each flow was accurately controlled by calibrated primary digital Mass Flow Controllers (MFCs, Bronkhorst, F-201CV, ΔΦ = 0.005Φ + 0.001 max scale). Equal gas flows were sent to the anode and cathode, from the centre of the cell and spreading outwards. The cell was heated up to 600 °C at a rate of 25 °C/h under ambient atmosphere then purged with pure nitrogen. The reduction of the nickel anode was performed with 5 v/v% of H_2_ in N_2_ for 22 h, before ramping up and down the O_2_ content of the gas. More specifically, both the anode and cathode were constantly fed with a primary mixture of 10.5 sccm of H_2_ and 198.5 sccm of N_2_. Oxygen was progressively added to this primary mixture until the ratio O_2_-to-H_2_ reached the value of 2, always keeping the exact same conditions on both sides of the SOFC. The partial pressures in the ex-situ configuration were several orders of magnitude higher than the ones achievable in the ETEM. The cell voltage was measured between the two gold meshes and the temperature was measured with a K-type thermocouple placed as close as possible to the cell (about 1 mm). Both signals were recorded online with a data logger (Fluke, Hydra).

### Supplementary information

Supplementary Information

### Source data

Source Data

## Data Availability

ETEM data used to make Figs. 2–4 are available at 10.5281/zenodo.8414459. Source data are provided with this paper.
