# Supplementary material for: Operando analysis of a solid oxide fuel cell by environmental transmission electron microscopy
Source: Nat Commun. 2023 Dec 2;14:7959. doi: 10.1038/s41467-023-43683-4 (PMC10693604; doi:10.1038/s41467-023-43683-4)
Supplement: Supplementary file 1 — Supplementary Information [file 41467_2023_43683_MOESM1_ESM.pdf]

Operando analysis of a solid oxide fuel cell by environmental transmission electron microscopy

Q. Jeangros,[1,2,*] M. Bugnet,[3] T. Epicier,[3,4] C. Frantz,[5] S. Diethelm,[5] D. Montinaro,[6] E. Tyukalova,[7] Y. Pivak,[8] J. Van herle,[5] A. Hessler-Wyser,[1] M. Duchamp[7,9,*]

[1] Photovoltaics and Thin-Film Electronics Laboratory (PVLab), École Polytechnique Fédérale de Lausanne (EPFL), Rue de la Maladière 71b, 2002 Neuchâtel, Switzerland.

[2] Centre Suisse d'Electronique et de Microtechnique (CSEM), Jaquet-Droz 1, 2002 Neuchâtel, Switzerland

[3] Univ Lyon, CNRS, INSA-Lyon, UCBL, MATEIS, UMR 5510, 69621 Villeurbanne, France

[4] Univ Lyon, UCBL, IRCELYON, UMR CNRS 5256, F-69626 Villeurbanne, France

[5] Group of Energy Materials (GEM), École Polytechnique Fédérale de Lausanne (EPFL), Rue de l'Industrie 17, 1951 Sion, Switzerland.

[6] SolydEra S.p.A., 38017, Mezzolombardo, Italy

[7] Laboratory for *in situ* & *operando* Electron Nanoscopy, School of Materials Science and Engineering, Nanyang Technological University (NTU), 50 Nanyang Avenue, Singapore 63737.

[8] DENSsolutions, Informaticalaan 12, 2628 ZD Delft, The Netherlands

[9] MajuLab, International Joint Research Unit UMI 3654, CNRS, Université Côte d'Azur, Sorbonne Université, National University of Singapore, Nanyang Technological University, Singapore, Singapore

*Corresponding authors: Quentin Jeangros (quentin.jeangros@csem.ch) & Martial Duchamp (martial.duchamp@gmail.com)

Supplementary information

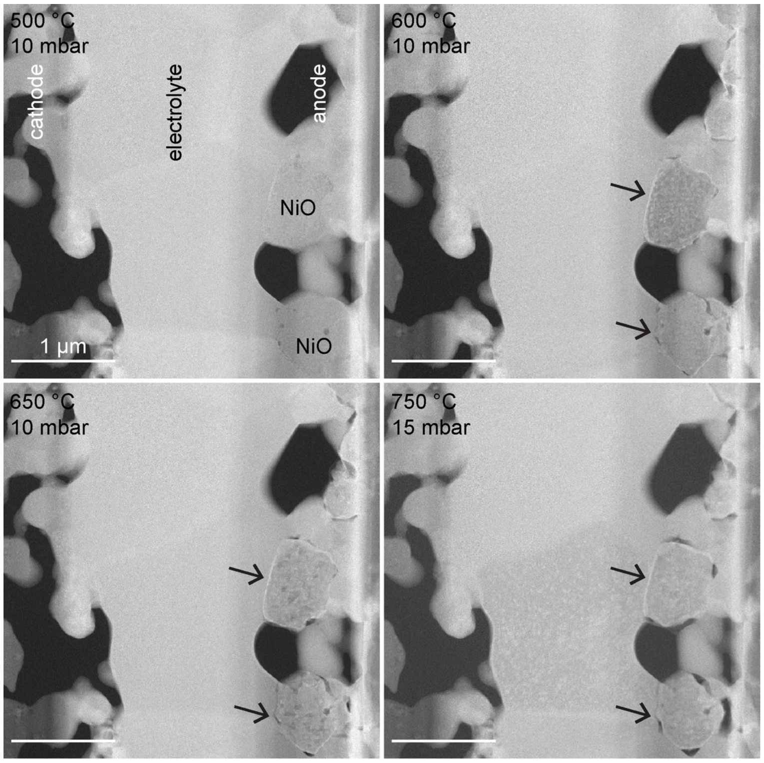

Figure S1: Sequence of STEM ADF of the first reduction of the SOFC lamella to activate the Ni catalyst. Arrows highlight the changes in morphology of the NiO grains, which shrink upon removal of oxygen. The orientation of the sample changed upon heating, leading to a difference in contrast (especially in the electrolyte YSZ phase).

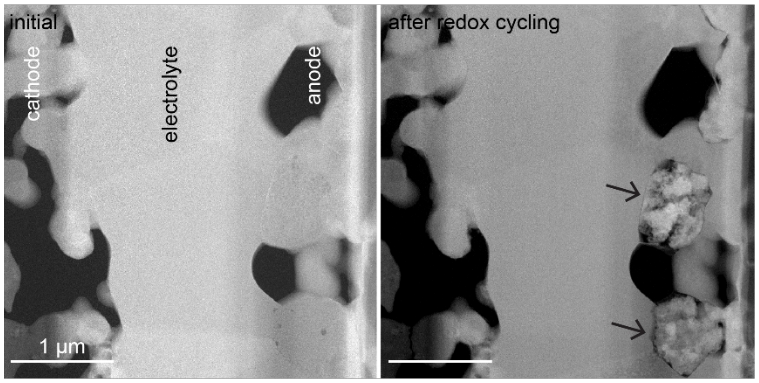

Figure S2: STEM ADF images of the SOFC lamella before and after multiple redox cycling and cell operation. The arrows show two characteristic Ni grains. The cathode remained unchanged during these experiments.

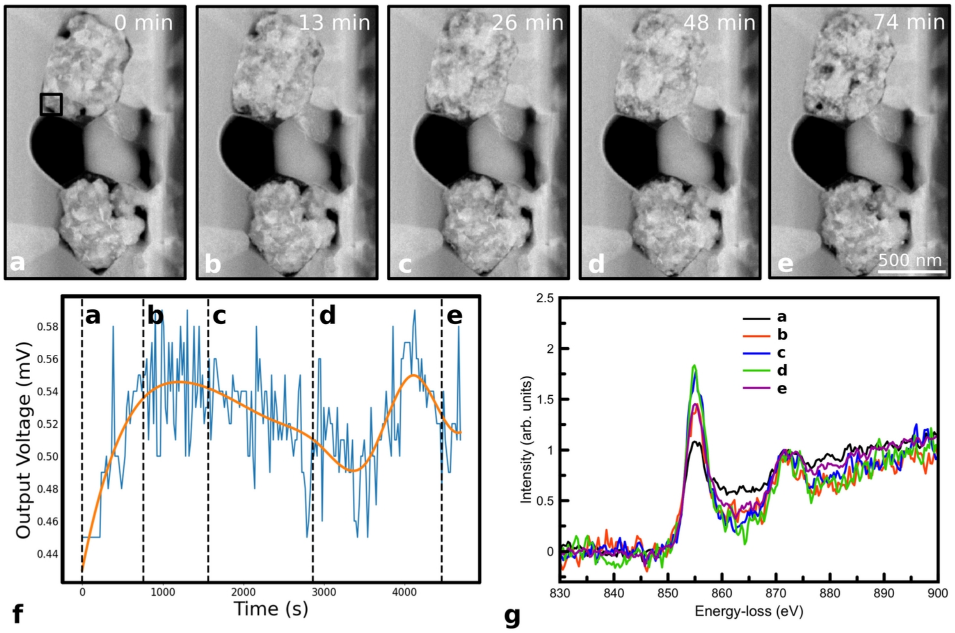

Figure S3: Microstructure, voltage and EELS data depending on O_2-to-H_2 ratio. a-e) STEM ADF micrographs of the Ni grains of the SOFC anode, first in 16 mbar of H_2/N_2, then exposed to an increasing amount of O_2 from 0 to 3000 s (a-d), before removing O_2 from the ETEM column 3000 s after the start of the experiment (e). A volume expansion occurs upon oxidation (a-d), while the Ni volume shrinks upon reduction (d-e). f) Corresponding evolution of the cell voltage with varying O_2-to-H_2 ratios (with and without filtering). The vertical dashed lines next to the letters a-e) correspond to the recording time of the corresponding micrographs a-e). g) EEL spectra of the Ni-$L_{2,3}$ edges recorded simultaneously with the STEM ADF micrographs shown in a-e) in the area marked by the black square in a). The spectra were background-subtracted, re-aligned in energy at the L_3 edge (855 eV) and normalised using the L_2 edge (872 eV). The increase in L_3-edge intensity with increasing O_2 concentration is visible, indicating an oxidation of Ni to NiO from a) to d).[61] The spectrum e) was measured after the removal of O_2 from the column and is representative of an intermediate NiO/Ni state, see main text for details.

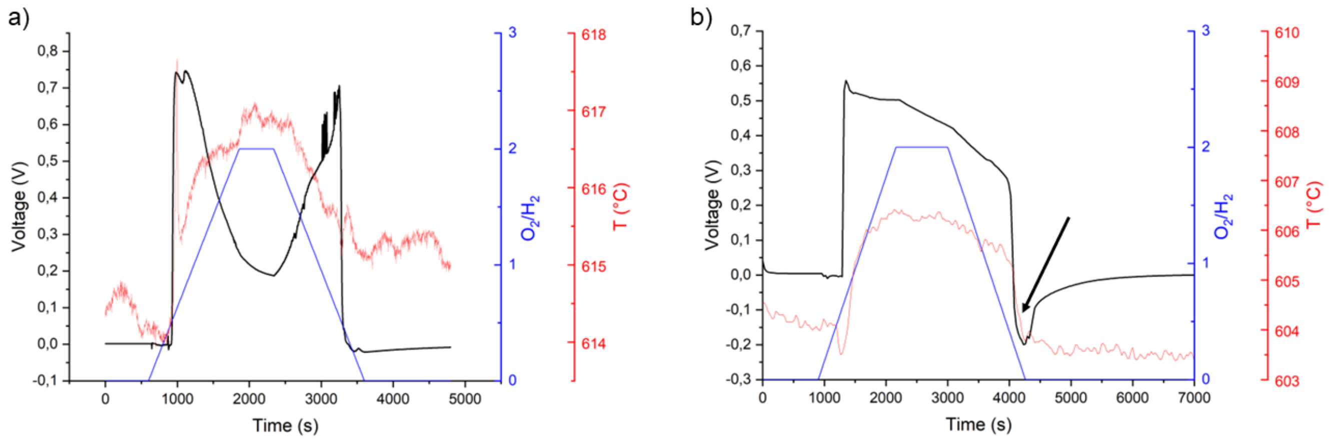

Figure S4: Starting with a metallic Ni anode in H_2 at around 600 °C, ex situ measurements of the evolution of the open circuit voltage and temperature together with the O_2-to-H_2 ratio (from calibrated MFCs) of a) a button cell featuring the same cathode-electrolyte-anode materials as those tested by ETEM, and b) a button half-cell electrolyte-anode with similar materials. In both cases, an Au mesh was used to contact the samples. The cathode-electrolyte-anode cell shown in a) operates around the stoichiometric O_2-to-H_2 ratio of 0.5 when ramping up and down the O_2-to-H_2 ratio. On the other hand, the electrolyte-anode half-cell exhibits different voltage trends, especially when decreasing the O_2-to-H_2 ratio: the voltage becomes negative meaning the anode acts as cathode with respect to the Au mesh in these conditions (see arrow).

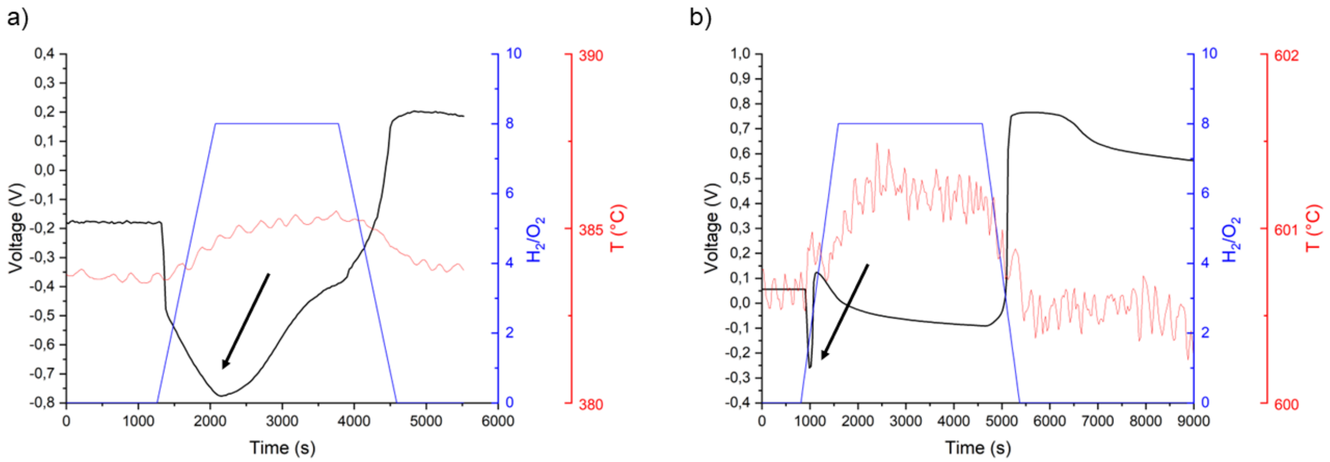

Figure S5: Starting with a NiO anode precursor in an O_2 atmosphere, ex situ measurements of the evolution of the OCV as a function of the H_2-to-O_2 ratio (from calibrated MFCs) of a button half-cell electrolyte-anode at a) 380 °C, and b) at 600 °C. In both cases, the voltage drops to negative values when NiO initially reduces to Ni (arrows), highlighting that the Au mesh acts as an anode in these conditions. The voltage increases to standard OCV values when Ni oxidises to NiO during the second stage of the experiment at 600 °C, in line with the initial OCV increase observed in Figure S4b.

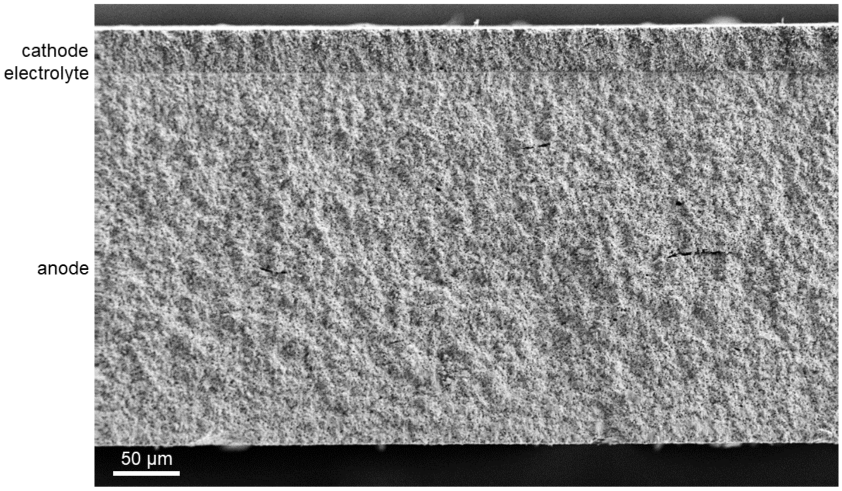

Figure S6: Cross-section SEM image of the cathode-electrolyte-anode button cell investigated in this study. 5-µm wide TEM lamellae were extracted by FIB from the cathode-electrolyte-anode interface.

Figure S7 shows a similar experiment to that performed in Figure S4b, with the exception that the half-cell was left at a constant O_2-to-H_2 ratio of 2 until reaching steady-state conditions. After an initial rise in OCV when ramping up the O_2-to-H_2 ratio (as in Figure S4b), the OCV then becomes negative as the anode oxidises completely, an effect not observed during the faster experiment shown in Figure S4b. Overall, the evolving microstructure and chemistry of the anode of the button cells lead to complex P_{O_2} trends. These effects are absent in the 200-nm thin TEM lamellae investigated in the ETEM as these samples feature large free surfaces exposed to the environment in comparison to the thickness of the sample.

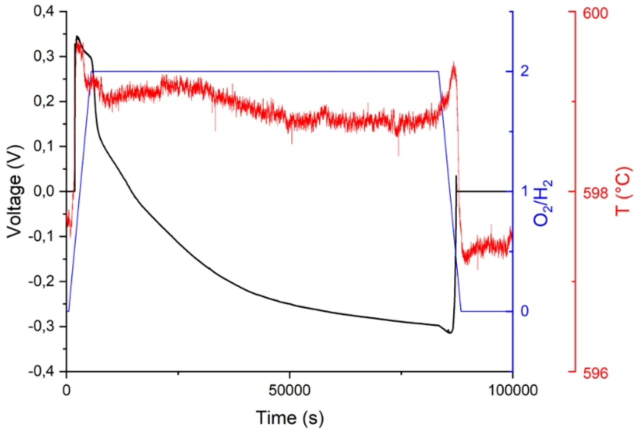

Figure S7: Starting with a metallic Ni anode in an H_2 atmosphere, ex situ measurements of the evolution of the open circuit voltage and temperature together with the O_2-to-H_2 ratio (from calibrated MFCs) of a button half-cell electrolyte-anode. An Au mesh was used to contact the sample. Compared to Figures S4 and S5, the aim here was to reach steady-state conditions at an O_2-to-H_2 ratio of 2. These results highlight the complex processes at play between local P_{O_2} and local Ni oxidation state, and how these evolve in time, eventually determining the OCV of the half-cell.

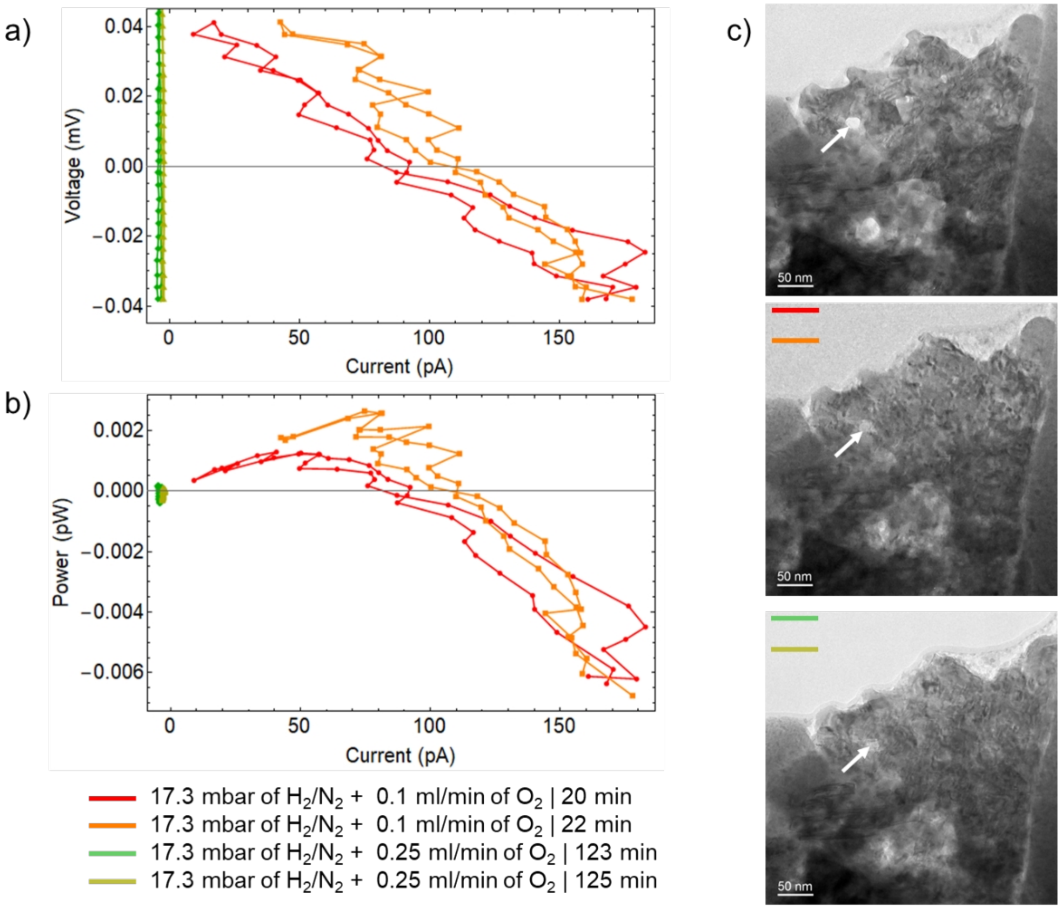

Figure S8: Current-voltage measurements of a SOFC sample inside an ETEM in a single-chamber configuration. a) Current-voltage properties of a cathode-electrolyte-anode cell measured in the ETEM when introducing a small partial pressure of O_2 (starting from a reduced Ni/YSZ anode in an H_2/N_2 atmosphere as in Figures 2-4) and b) corresponding power-current curves characteristics qualitatively similar to standard SOFCs until the system becomes too resistive. Corresponding TEM images are shown in c) (bottom rows) alongside an image taken prior to the introduction of O_2 in the microscope chamber (top row). The arrows highlight the oxidation of the Ni catalyst already after 20 min into the experiment (volume expansion filling a void in the Ni microstructure), meaning already before the system becomes highly resistive as likely fully oxidized (>120 min after the introduction of O_2).

Stack of images and movies of the full sequences of data shown in Figures 2,3 and 4 are provided as separate files on DOI:10.5281/zenodo.8414459.
